# Bromsulphalein Excretion in p-Dimethylaminoazobenzene-Treated Rats

**DOI:** 10.1038/bjc.1958.68

**Published:** 1958-12

**Authors:** J. S. Howell


### BROMSULPHALEIN EXCRETION IN *p*-DIMETHYLAMINOAZOBENZENE-TREATED RATS

J. S. HOWELL

*From the Department of Pathology and Cancer Research, University of Birmingham*

Received for publication September 23, 1958

In a previous paper (Howell, 1958) the inhibitory effect of copper acetate on the development of hepatic tumours in rats treated with *p*-dimethylaminoazobenzene (DMAB) was described. Liver function was assessed by means of the bromsulphalein (BSP) excretion test in the hope of obtaining some biochemical measure of the rate of progression of the liver changes and to determine whether a correlation could be found between the histological changes in the liver, the type of tumour and the result of the test. It was hoped to discover whether or not functional, as well as histological differences, existed between the rats given DMAB with and without copper acetate.

The BSP test was chosen from the many available tests of liver function for a variety of reasons. It has been shown by Mateer *et al.* (1947) to be a reliable screening test in man with chronic hepatitis, cirrhosis or metastatic carcinoma, and one which could be used to measure the rate of progression or regression of these disease processes. Treatment of the rat with DMAB causes gradual destruction of liver cells and a concomitant fibrosis, and its effects can be compared with those of chronic hepatitis and cirrhosis in man in this respect. As a method of investigating liver function in experimental animals, the value of the BSP test has been demonstrated by Drill and Ivy (1944) in dogs treated with carbon tetrachloride and by Casals and Olitsky (1946) in mice subjected to various types of hepatic injury. More recently its value has again been demonstrated by Koch-Weser, Farber and Popper (1951), Waldstein, Ettinger and Giges (1957) and by Aterman and Howell (1959). The test has advantages in that the technique is fairly simple, so that a large number of animals can be tested in a short time, and also that the dye can be detected in very small quantitites of serum. Furthermore, there is no evidence that injection of the dye causes additional damage to an already damaged liver (Mateer *et al.*, 1947).

### MATERIALS AND METHODS

Seventy albino rats of a heterozygous strain were used (Laboratory Animals Bureau Catalogue of Uniform Strains, No. 626, 1953; Birmingham strain). They were divided into 7 groups containing equal numbers of males and females. The dietary treatment of each of these groups (Tables I, II and III), together with the reasons for this arrangement have been given in a previous publication (Howell, 1958).

BSP tests were not undertaken before the 9th month of treatment. Tests were performed on many of the available animals during this month, again after 12 and 15 months, and at other times if it was thought that an animal had developed a hepatic tumour.

TABLE I.—*Groups* 5, 6 *and* 7. *Diets*

| Group | Number of rats: Male | Female | Diet |
|---|---|---|---|
| 5 | 5 | 5 | M + DMAB. |
| 6 | 5 | 5 | M + DMAB + Cu ac. |
| 7 | 5 | 5 | M + DMAB + Fe cit. |

M = Maize, DMAB = 0·09 per cent*p*- Dimethylaminoazobenzene, Cu ac = 0·5 per cent Copper acetate, Fe cit = 2·0 per cent Ferric citrate.

TABLE II.—*Groups* 8, 9, 10 *and* 11. *Diets : DMAB and Copper Acetate Components Given Separately*

| Group | Number of rats: Male | Female | Diet | Number of days per week |
|---|---|---|---|---|
| 8 | 5 | 5 | M + DMAB + Fe cit | 4 |
| | | | M + Cu ac | 3 |
| 9 | 5 | 5 | M + DMAB + Fe cit | 5 |
| | | | M + Cu ac | 2 |
| 10 | 5 | 5 | M + DMAB + Fe cit | 6 |
| | | | M + Cu ac | 1 |
| 11 | 5 | 5 | M + DMAB + Fe cit | 4 |
| | | | CP | 3 |

M = Maize, CP = Cube powder, Fe cit = Ferric citrate, Cu ac = Copper acetate, DMAB = 0·09 per cent *p*-Dimethylaminoazobenzene.

TABLE III.—*Groups* 8, 9, 10 *and* 11. *To Show Days of Week on which DMAB or Copper Acetate Was Given*

| Group | Monday | Tuesday | Wednesday | Thursday | Friday | Saturday | Sunday |
|---|---|---|---|---|---|---|---|
| 8 | Cu ac | DMAB | Cu ac | DMAB | Cu ac | DMAB | DMAB |
| 9 | ,, | ,, | DMAB | ,, | ,, | ,, | ,, |
| 10 | DMAB | ,, | ,, | ,, | ,, | ,, | ,, |
| 11 | CP | ,, | CP | ,, | CP | ,, | ,, |

Basal diet maize, except where CP is indicated.

During the test, with the animal anaesthetised, careful abdominal palpation enabled tumours to be detected at an earlier stage than was otherwise possible, and when a tumour was found the animal was usually killed immediately after completion of the test. Some animals without tumours died shortly after the completion of the test, as an effect of the test itself. Post-mortem examinations were made and blocks of liver from tumorous and non-tumorous areas were preserved. Sections were stained, and liver damage was assessed according to the methods and criteria previously described (Howell, 1958).

The technique of the BSP test has been described in detail in previous publications (Howell, 1957 ; Aterman and Howell, 1959). All rats were fasted over-night and then weighed to the nearest gram. A solution of BSP (G. Gurr and Sons) was injected into the exposed jugular vein, dosage being calculated on 25 mg./kg. body weight. Exactly 30 minutes after completion of the injection about 2·0 ml. of blood was withdrawn by cardiac puncture. The blood was centrifuged and the colour of the dye was developed in the serum by the addition of 10 per cent sodium hydroxide. The amount of BSP present in the serum was estimated colorimetrically in the Unicam spectrophotometer using blanks prepared from saline and from acidified serum. It should be mentioned that certain rats, with clinically obvious tumours, were found to have icteric sera, but the results of the test in these animals were ignored since it was thought that they were likely to be fallacious.

### RESULTS

### *Normal Rats (BSP Present in Serum 30 Minutes After Injection)*

This determination was made on 15 rats with an average weight of 300 g. The mean value at 30 minutes was 0·322 mg. per 100 ml. serum (range = 0·58 to complete excretion, S.D.= 0·18). It was assumed therefore that BSP in the serum of a normal rat at 30 minutes would be very unlikely to exceed 1 mg./100 ml. serum, and values of this figure and above were regarded as abnormal; values below 1 mg./100 ml. serum were regarded as normal.

### *DMAB-treated Rats*

*Group 5. Maize and DMAB*

The first tests were made after 9 months in 4 of the 5 animals surviving at this time. Three of these, including one with a palpable liver tumour, gave normal values, i.e. less than 1 mg. /100 ml. serum. The 4th animal, also with a tumour, had abnormal BSP retention. After 10 months only 2 animals remained, both with liver tumours, and abnormal BSP retention was found; they had given normal BSP values at 9 months.

*Group 6. Maize, DMAB and copper acetate*

All 8 available animals were tested at 10 months. None had palpable liver tumours and all had normal BSP values. After 15 months the 2 surviving rats were again tested. Neither rat had a liver tumour and both gave normal BSP values.

*Group 7. Maize, DMAB and ferric citrate*

Four of the 5 available rats were tested at 9 months. Three of them had palpable liver tumours, of which one had abnormal BSP retention. The tumour-free animal gave a normal result. During the 10th month an animal was tested with an abnormal result; it had a palpable liver tumour. At 12 months the sole survivor was retested, by which time a tumour had developed and the result was abnormal.

*Group 8. (1) Maize, DMAB and ferric citrate, 4 days per week; (2) maize and copper acetate, 3 days per week*

Three of the available 9 animals were tested at 9 months; none had liver tumours, and the result was normal in all 3. Six of the available 8 were retested at 12 months, all with normal results, none having liver tumours. Six were again tested at 15 months, all with normal results, one of the animals having a liver tumour at this time.

*Group* 9. (1) *Maize, DMAB and ferric citrate,* 5 *days per week ;* (2) *maize and copper acetate,* 2 *days per week*

Of 6 available rats, 4 were tested at 9 months. Three, including 2 with tumours, gave normal values ; the remaining animal, also with a tumour, gave an abnormal result. The 3 surviving animals in the group were retested at 11 months and 2 gave normal results ; neither having tumours ; the third had a tumour and gave an abnormal result.

*Group* 10. (1) *Maize, DMAB and ferric citrate,* 6 *days per week ;* (2) *maize and copper acetate,* 1 *day per week*

Three rats out of the 9 available were tested at 9 months. Abnormal results were obtained in 2, both of which had tumours, but the 3rd animal, also with a tumour, gave a normal result. After 12 months all 6 survivors were tested and abnormal results were obtained in 2 ; both had tumours. The 4 animals giving normal values included 2 with tumours. One of the rats tested at 12 months with a normal result was retested at 13 months, by which time a tumour had developed, and abnormal BSP retention was found.

*Group* 11. *Maize, DMAB and ferric citrate,* 4 *days per week ; rat cube,* 3 *days per week*

One rat at 9 months and another at 11 months were tested ; both gave normal results, neither having liver tumours. After 12 months all 6 available animals were tested, and all gave normal results. After 15 months the same animals were retested, and again all had normal results, although at this time 3 of them had liver tumours.

### *Correlation of BSP Test with Histology*

1. *Normal BSP test*

(a) *Microscopical changes in liver of animals without tumours.*—Material is available from 6 rats, which died either at the conclusion or within one week of the test. Microscopically all showed severe liver cell damage and 3 showed varying degrees of regenerative hyperplasia. Marked periportal chronic inflammatory cell and macrophage infiltration was observed associated with proliferation of bile ducts, but in only one rat was cirrhosis seen which was early in type (Table IV).

(b) *Microscopical changes in liver of animals with tumours.*—There were 10 rats in this group, in 5 of which the tumour was confined to one or two lobes ; in the

TABLE IV.—*Rats Without Tumours Dying Shortly After BSP Test. BSP Values Correlated with Cirrhosis and Liver Weight*

| Rat No. | Dietary group | BSP (mg./100 ml. serum) | Liver weight (g.) | Cirrhosis |
|---|---|---|---|---|
| 1 | 11 | 0·72 | 8 | — |
| 2 | 11 | 0·62 | 10 | — |
| 3 | 10 | 0·61 | 11 | — |
| 4 | 8 | 0·71 | 10 | — |
| 5 | 6 | 0·66 | 10 | ++ |
| 6 | 6 | 0·53 | 11 | — |

Mean weight of liver = 10 g.
— = no cirrhosis. ++ = early cirrhosis.

remaining 5, tumours of varying size were scattered throughout all lobes of the liver. The types and combinations of tumours are set out in Table V. Sections of non-tumorous liver from these animals invariably revealed varying degrees of liver cell damage. Cirrhosis was present in 7 animals, varying from incipient to advanced in type, but was absent in the remaining 3. All 10 animals showed architectural abnormalities of the liver lobule, some showing definite regeneration nodules. All showed marked periportal chronic inflammatory cell infiltration and bile duct hyperplasia.

TABLE V.—*Normal BSP Retention Correlated with Tumour Type, Cirrhosis and Liver Weight*

| Rat No. | Dietary group | BSP (mg/100 ml. serum) | Liver weight (g.) | Tumour type | Cirrhosis |
|---|---|---|---|---|---|
| 1 | 11 | C.E. | 18 | A | — |
| 2 | 11 | 0·26 | 14 | H + A | ++ |
| 3 | 11 | C.E. | 18 | H + A | — |
| 4 | 10 | „ | 12 | A | + |
| 5 | 10 | „ | 30 | A + M | ++ |
| 6 | 9 | „ | 12 | A + C | ++ |
| 7 | 9 | 0·27 | 11 | A | ++ |
| 8 | 8 | C.E. | 35 | H + A | — |
| 9 | 7 | 0·08 | 18 | M | +++ |
| 10 | 5 | 0·68 | 15 | H + M | +++ |

Mean weight of liver = 18·3 g., C.E. = Complete excretion.
H = hepatoma, A = cystadenoma, M = hepatoma + cholangiocarcinoma ,C = cholangiofibrosis.
+ = Incipient cirrhosis, ++ = early cirrhosis, +++ = advanced cirrhosis, — = no cirrhosis.

### 2. *Abnormal BSP test*

*Microscopical changes in the liver.*—Gross examination of the liver of the 11 rats with tumours and abnormal BSP retention showed that, with the exception of a single rat, the liver was diffusely involved by tumour. By this is meant that tumour nodules were found in all lobes, and whilst one lobe might show a larger tumour than the others, no definite location of a " primary " tumour could be given. The exception was an animal with a tumour confined to the right lobe of the liver. The incidence of the various types and combinations of tumours are given in Table VI. All the rats in this group showed irregular granularity of the surface of the non-tumorous parts of the liver. Microscopic examination of these areas invariably showed regenerative hyperplasia, increased cellularity of the portal tracts and bile duct proliferation. Cirrhosis was present in all these animals, being advanced in 5 and early in the remaining 5.

### DISCUSSION

The results of the BSP test in DMAB-treated rats were disappointing and failed to reveal differences between animals treated with DMAB alone and those treated with DMAB and copper acetate. Furthermore, the test did not give any information regarding the severity of the pre-neoplastic changes in the liver. There were 6 animals that were tested after prolonged treatment with DMAB but which had not produced tumours. BSP excretion in these animals was entirely normal even though they all showed a severe degree of liver cell damage associated with regenerative hyperplasia, bile duct hyperplasia and in one instance early cirrhosis.

TABLE VI.—*Abnormal BSP Retention Correlated with Tumour Type Cirrhosis and Liver Weight*

| Rat No. | Dietary group | BSP (mg./100 ml. serum) | Liver weight (g.) | Tumour type | Cirrhosis |
|---|---|---|---|---|---|
| 1 | 5 | 1·35 | 10 | H + A | +++ |
| 2 | 5 | 2·31 | 30 | H | ++ |
| 3 | 5 | 2·71 | 25 | H | +++ |
| 4 | 7 | 2·80 | 45 | M | +++ |
| 5 | 7 | 1·27 | 65 | M + C | +++ |
| 6 | 7 | 1·37 | 12 | H | ++ |
| 7 | 9 | 3·31 | 65 | M | ++ |
| 8 | 9 | 2·15 | 30 | H + C | ++ |
| 9 | 10 | 1·27 | 30 | M | ++ |
| 10 | 10 | 1·57 | 40 | M + A | +++ |
| 11 | 10 | 1·00 | 75 | M + A + C | ++ |

Mean weight of liver = 38·81 g.
H = hepatoma, A = cystadenoma, M = hepatoma + cholangiocarcinoma, C = cholangiofibrosis.
+ = Incipient cirrhosis, ++ = early cirrhosis, +++ = advanced cirrhosis.

It was not until tumours had developed that the result of the test sometimes, but not always, became abnormal. Thus 11 rats with tumours had abnormal dye retention, but in 10, also with tumours, excretion was normal; there were no major histological differences between these two groups of animals. No particular type of tumour predominated in either group, although the tumours which occur earliest in the course of DMAB carcinogenesis, e.g. cystadenomata, tended to be more frequent in the group with normal BSP excretion. Non-tumorous liver from both groups invariably showed severe damage, with necrosis of liver cells, regenerative hyperplasia, bile duct proliferation, and in all but 3 animals early or advanced cirrhosis. It is of interest that the 3 animals without cirrhosis were able to excrete the dye completely.

Although there were no major histological differences between the animals with and without abnormal dye retention there was a marked difference in the weight of the liver which could be correlated with the result of the test. The mean weight of the liver of the 10 animals with tumours, but with normal BSP excretion was 18·3 g. (Table V), but the mean weight of the liver of the 11 rats with BSP retention and tumours was 38·8 g. (Table VI). This difference is statistically significant ($0{\cdot}02 > P > 0{\cdot}01$). The tumours in the former group were smaller, more " focal " in distribution and were more often confined to one lobe of the liver than those in the latter group. The weight of the liver is a function of the amount of tumour present and the amount of destruction of normal liver parenchyma. The difference in liver size was also reflected in the ease with which a diagnosis of hepatic tumour development was made. The rats in the group with the heavier livers had obvious tumours, whereas the tumours in the other group were only found when the animals were anaesthetised.

Several points of interest arise from this study. Even in the presence of much chronic liver damage, associated with regenerative hyperplasia and the development of cirrhosis the ability of the liver to excrete BSP is unimpaired for a considerable time. Even when a tumour develops it is not until by growth and replacement of a large volume of liver tissue by tumour that the test becomes abnormal. It is possible that abnormal excretion is dependent upon the ratio of tumour to non-neoplastic liver in that this may determine the proportion of the blood supply shunted away from functional liver tissue to non-functional tumour. However, even the presence of quite large tumours does not necessarily mean that BSP excretion will be abnormal since 5 rats with tumours, including 2 with livers weighing 30 g. or more were able to excrete the dye completely (Table V).

The reason why this test remains normal for such a considerable time is not clear. In the initial stages of the experiment considerable doubt was felt about the validity of the test because of the normal results obtained. A study was therefore undertaken of BSP excretion in 15 hypothyroid rats in which the main pathological feature was fatty change in the liver. BSP excretion was impaired in many of these animals until thyroid extract was given, when it became normal; this was followed by renewed impairment when thyroid extract was discontinued (Aterman and Howell, 1959). Hence it was concluded that the method of testing and the calculation of results of BSP excretion were reliable.

The value of liver function tests in human hepatic cancer is disputed. Berman (1951) in his monograph states that most liver function tests devised thus far are of doubtful value. Spellberg (1954) in his book states " that liver function tests are variable and depend to a great extent on an underlying cirrhosis ", but also " that the BSP is likely to be increased ". Sherlock (1958) in her text-book states that the BSP test is positive in hepatic cancer. Spatt and Grayzel (1948), Schupback and Chappell (1952), Galluzi *et al.* (1953) and Bersohn (1957) all found abnormal BSP excretion in their cases of primary hepatic cancer. But Holley and Pierson (1948) concluded that liver function tests as a rule were disappointing as a method of diagnosis of primary carcinoma of the liver, and that damage must be extensive before any appreciable change can be detected in the function tests. In cases of secondary carcinoma involving the liver there is greater uniformity of opinion and several workers have agreed that the BSP test is of value in diagnosis, e.g. Paulson and Wyler (1942), Thomas and Zimmerman (1952), Shay and Siplet (1954) and Sherlock (1958) and it has been suggested that the results are abnormal because of interference with blood supply caused by the secondary deposits. However, this does not explain the discrepancy of the results in primary carcinoma since the cirrhosis usually associated with this condition would be expected to cause greater interference with normal blood supply.

The clinical diagnosis of primary hepatic cancer in man is notoriously difficult and usually when the diagnosis is made and liver function tests have been undertaken the tumour is large and much destruction of liver tissue has occurred. It is concluded that there is some correlation between the results of BSP excretion in man and in the experimental animals in that the result of the test depends to a great extent on the size of the tumour and on the amount of tumour replacement of liver parenchyma. It is also concluded that the BSP test is of little value in assessing functional impairment of the rat's liver due to DMAB in the pre-neoplastic stages.

### SUMMARY

Liver function has been assessed by means of the BSP excretion test in rats treated with DMAB. The results of the test, regardless of the severity of the liver damage, were entirely normal until a tumour had developed. Ten rats with tumours had normal BSP excretion and 11 rats, also with tumours, had abnormal BSP excretion. Abnormal BSP excretion is independent of tumour type and underlying liver damage, but apparently depends upon the size of tumour and the degree of replacement by tumour of liver tissue. It is suggested that these results conform with those obtained in human cases of primary hepatic cancer.

I am indebted to Professor J. W. Orr and Dr. D. Hamer for valuable help and advice throughout this investigation. I am also indebted to the Birmingham Branch of the British Empire Cancer Campaign and to the Medical Research Fund of the United Birmingham Hospitals for support of this work.

### REFERENCES

Aterman, K. and Howell, J. S.—(1959) *Lab. Invest.* (in press).
Berman, C.—(1951) ' Primary Carcinoma of the Liver '. London (H. K. Lewis & Co. Ltd.), p. 37.
Bersohn, I.—(1957) *S. Afr. med. J.*, **31**, 828.
Casals, J. and Olitsky, P. K.—(1946) *Proc. Soc. exp. Biol. N.Y.*, **63**, 383.
Drill, V. A. and Ivy, A. C.—(1944) *J. clin. Invest.*, **23**, 209.
Galluzi, N. J., Weingarten, W., Regan, F. D. and Doerner, A. A.—(1953) *J. Amer. med. Ass.*, **152**, 15.
Holley, H. L. and Pierson, G.—(1948) *Amer. J. Med.*, **5**, 561.
Howell, J. S.—(1957) M.D. Thesis, University of Birmingham.—(1958) *Brit. J. Cancer*, **12**, 594.
Koch-Weser, D., Farber, E. and Popper, H.—(1951) *Arch. Path.* (*Lab. Med.*), **51**, 498.
Mateer, J. G., Baltz, J. I., Steele, H. H., Brouwer, S. W. and Colvert, J. R.—(1947) *J. Amer. med. Ass.*, **133**, 909.
Paulson, M. and Wyler, C. I.—(1942) *Ann. intern. Med.*, **16**, 872.
Schupback, H. J. and Chappell, R. B.—(1952) *Arch. intern. Med.*, **89**, 436.
Shay, H. and Siplet, H.—(1954) *J. Lab. clin. Med.*, **43**, 741.
Sherlock, S.—(1958) ' Diseases of the Liver and Biliary System '. 2nd ed. Oxford (Blackwell Scientific Publications), pp. 573, 585.
Spatt, S. D. and Grayzel, D. M.—(1948) *Amer. J. Med.*, **5**, 570.
Spellberg, M. A.—(1954) ' Diseases of the Liver '. New York (Grune and Stratton), p. 142.
Thomas, L. J. and Zimmerman, H. J.—(1952) *J. Lab. clin. Med.*, **39**, 882.
Waldstein, S. S., Ettinger, R. H. and Giges, B.—(1957) *Metabolism*, **6**, 134.


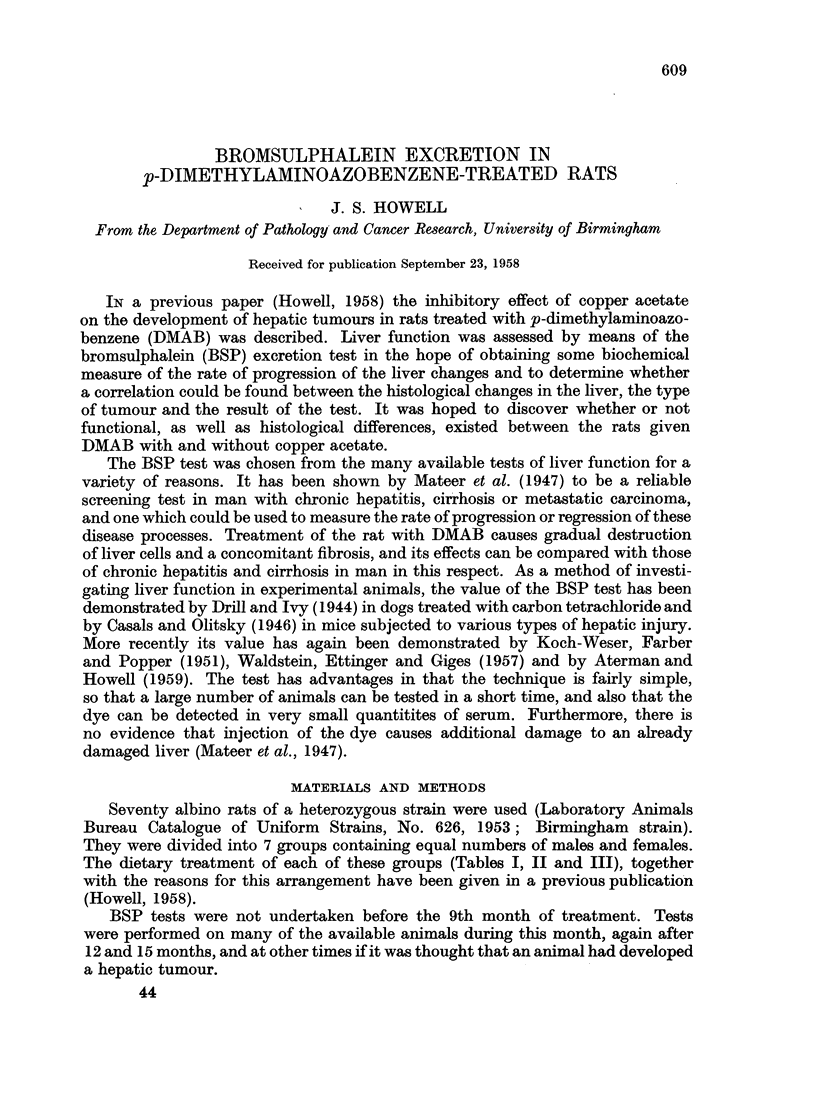

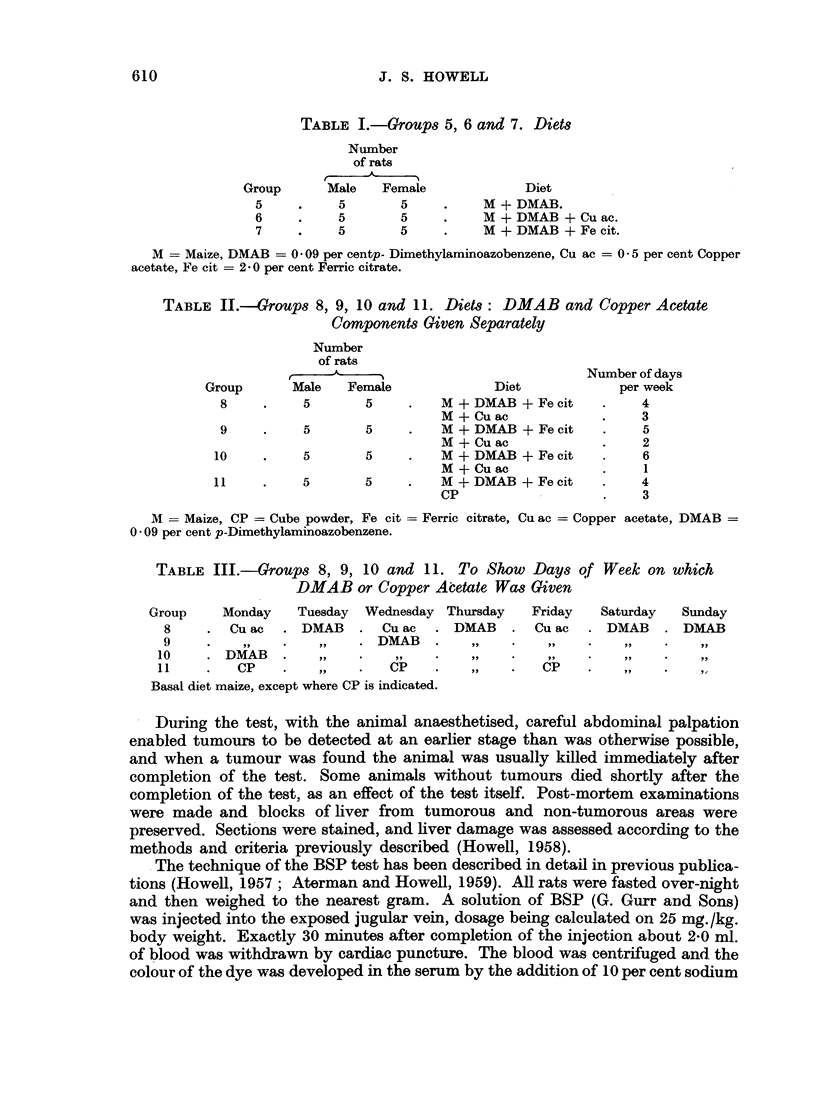

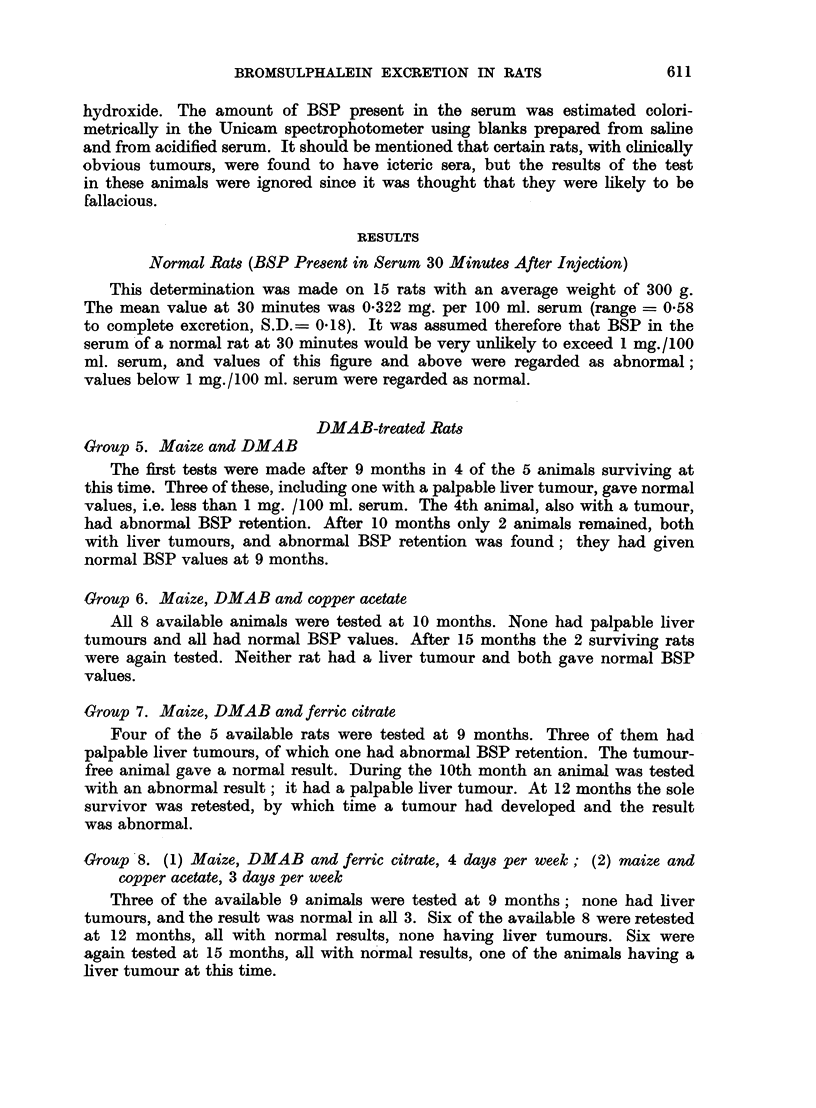

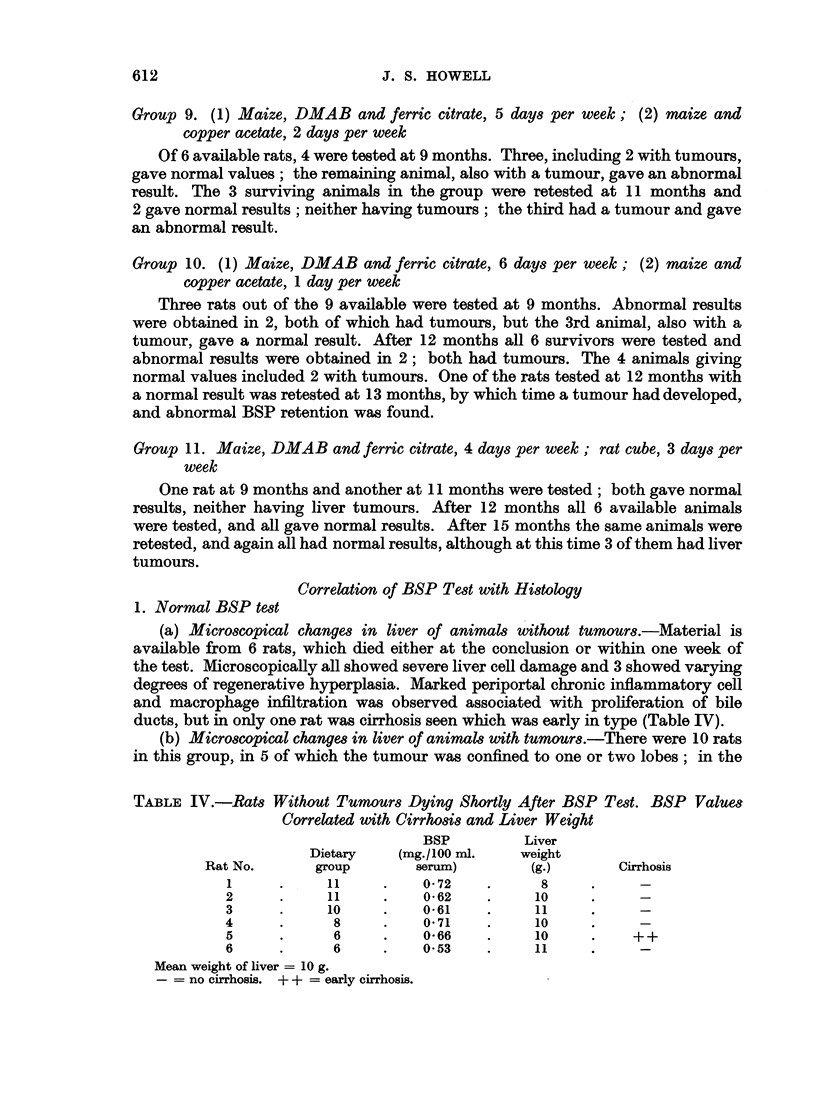

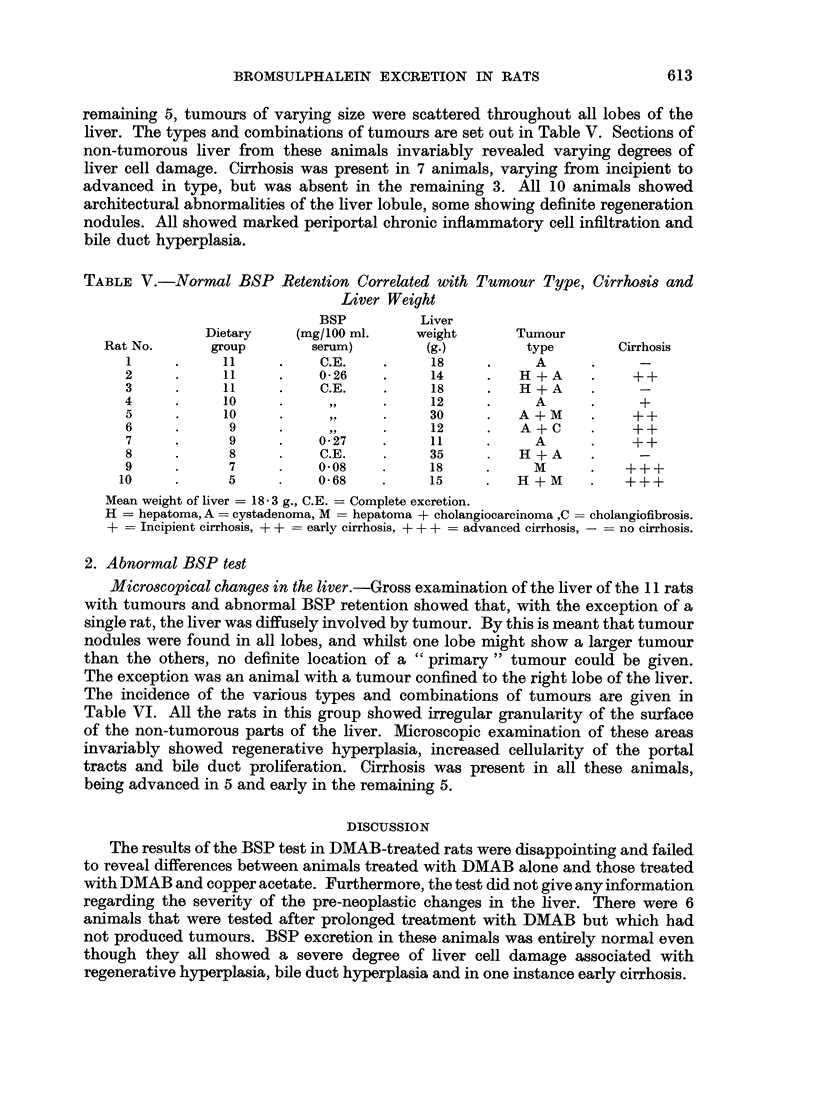

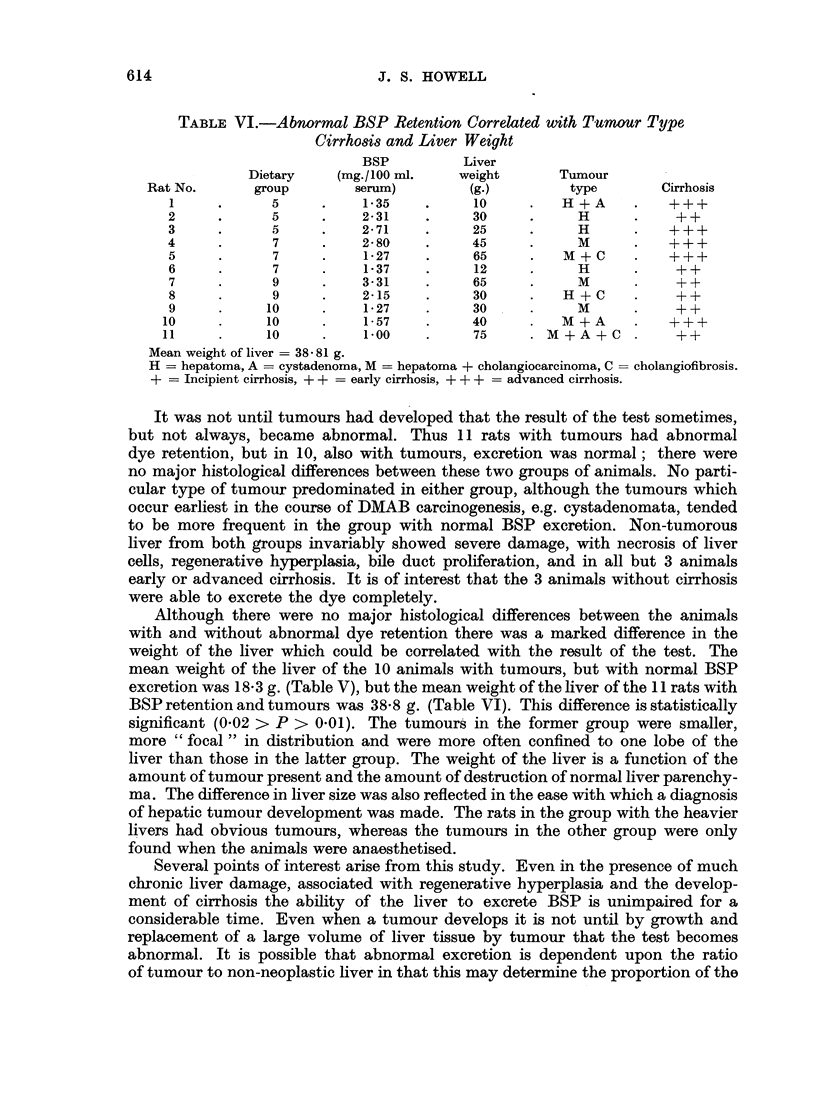

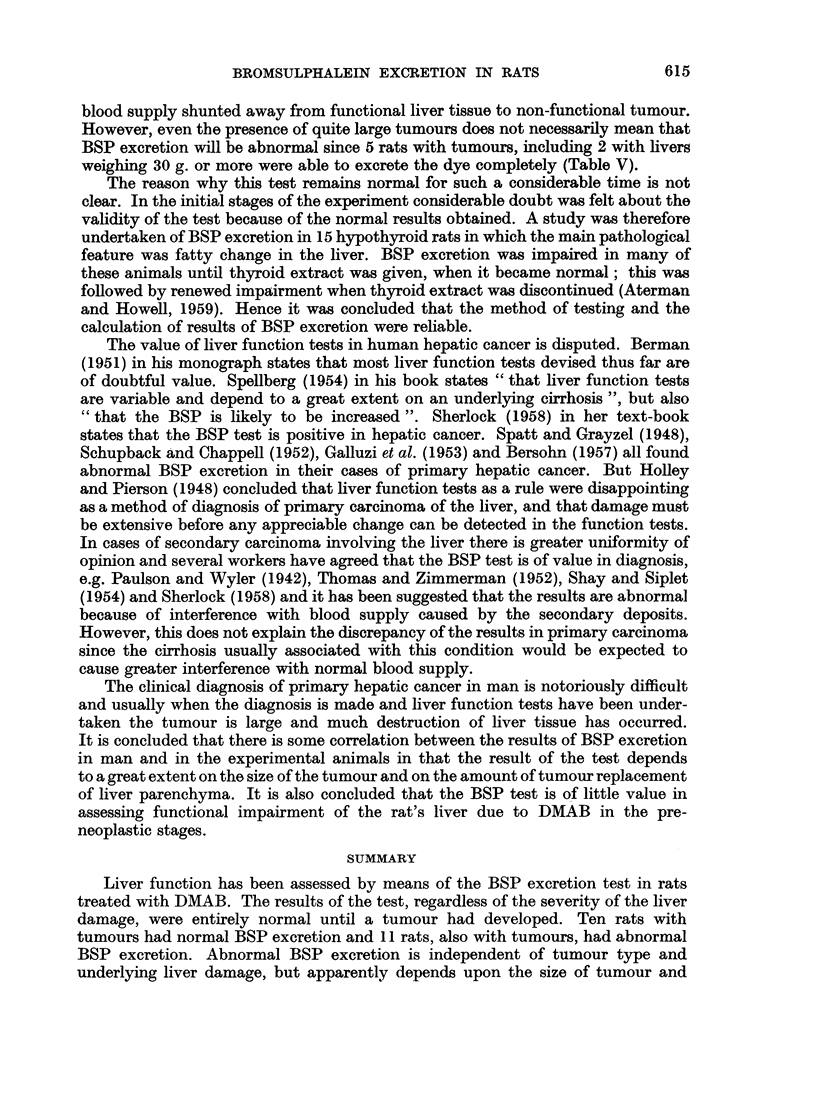

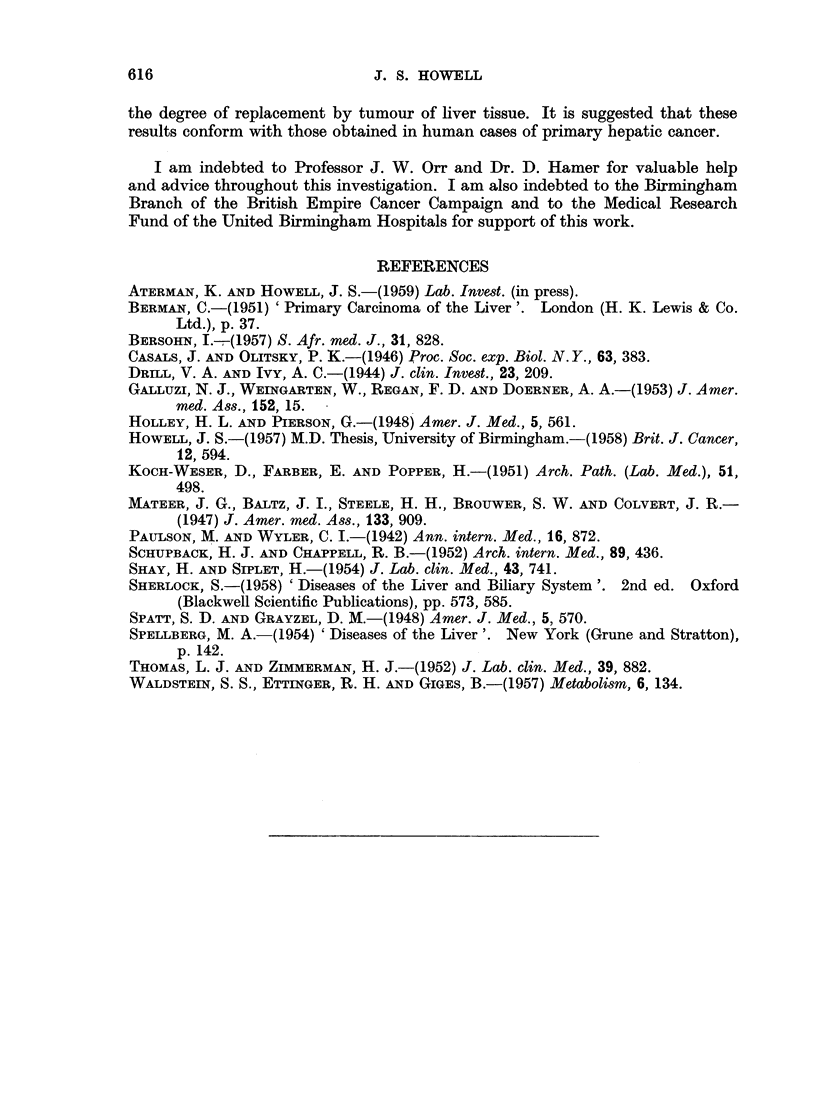


## References

[OCR_00521] BERSOHN I. (1957). Liver function tests in primary carcinoma of the liver in the South African Bantu.. S Afr Med J.

[OCR_00522] Drill V. A., Ivy A. C. (1944). COMPARATIVE VALUE OF BROMSULPHALEIN, SERUM PHOSPHATASE, PROTHROMBIN TIME, AND INTRAVENOUS GALACTOSE TOLERANCE TESTS IN DETECTING HEPATIC DAMAGE PRODUCED BY CARBON TETRACHLORIDE.. J Clin Invest.

[OCR_00532] HOWELL J. S. (1958). The effect of copper acetate on p-dimethylaminoazobenzene carcinogenesis in the rat.. Br J Cancer.

[OCR_00545] SHAY H., SIPLET H. (1954). The value of serum alkaline phosphatase determination and bromsulphalein test in the diagnosis of metastatic cancer of the liver.. J Lab Clin Med.

[OCR_00557] THOMAS L. J., ZIMMERMAN H. J. (1952). The pattern of abnormality of liver function in metastatic carcinoma.. J Lab Clin Med.

[OCR_00559] WALDSTEIN S. S., ETTINGER R. H., GIGES B. (1957). Abnormal glucose tolerance in experimental choline-deficient fatty liver.. Metabolism.

